# A Rare Case of Petrified Ear

**DOI:** 10.1155/2012/410601

**Published:** 2012-10-15

**Authors:** Kathryn E. Buikema, Erin G. Adams

**Affiliations:** ^1^Kimbrough Ambulatory Care Center, Fort George G. Meade, MD 20755, USA; ^2^Department of Dermatology, Walter Reed National Military Medical Center, 8901 Wisconsin Avenue, Bethesda, MD 20889, USA

## Abstract

Calcification or ossification of the auricle, also referred to as petrified ear, is a rare diagnosis in dermatology. In medical literature, it has most often been attributed to trauma, hypothermia and frostbite, or hypercalcemia secondary to a metabolic or endocrine disorder, such as Addison's disease. Here, we report the clinical and radiologic findings of a 79-year-old African American male whose unilateral petrified auricle was an incidental finding. He had a preceding history of hyperparathyroidism and subsequent hypercalcemia treated with a subtotal parathyroidectomy three years prior to presentation. In addition to laboratory analysis, a history and physical examination was performed which revealed no other signs of hypercalcemia. Radiologic studies demonstrated partial ossification of the external auricular cartilage on the left side. The patient was diagnosed with the rare occurrence of a petrified ear. In light of this case, we provide a discussion concerning the possible etiologies of this diagnosis including appropriate patient evaluation and possible treatment recommendations.

## 1. Introduction

Petrified ear, whether due to calcification or ossification, is a rare diagnosis. Generally patients are asymptomatic, and the diagnosis is made incidentally. These patients often have endocrine or metabolic disorders that lead to hypercalcemia and ectopic calcification or ossification. In other cases, patients recall trauma to the ear or an episode of frostbite. Greater awareness of this diagnosis would be beneficial for all practitioners, especially considering its association with potentially serious and life-threatening endocrinopathies, such as cortisol deficiency.

## 2. Case Report

We present a case of a 79-year-old African American male referred from his internist for evaluation of a rigid left ear. The patient reported increased difficulty in fitting his hearing aid in his left ear over the preceding year. He denied history of wrestling, boxing, headset use, frostbite, or other trauma to the ear. He denied other systemic symptoms. His medical history revealed persistent hypercalcemia five years prior to presentation which had been attributed to hyperparathyroidism. He was treated with a subtotal parathyroidectomy three years prior to our evaluation which resulted in normalization of his serum calcium and parathyroid hormone levels.

**Figure 1:**
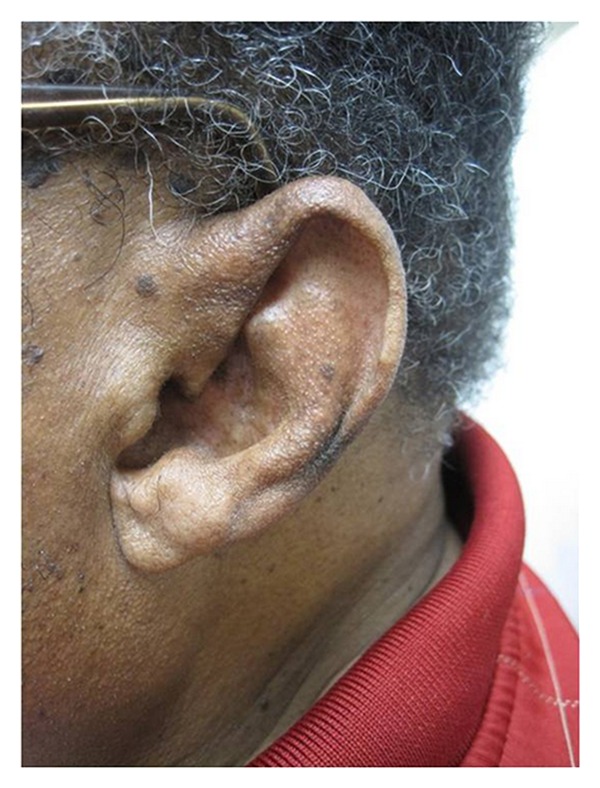
Left ear demonstrating no gross abnormalities.

On physical examination, the left anterior helix was markedly rigid and could not be folded. The superior helix to the crus of the helix was hyperpigmented and seemed bound to the crura of the antihelix (Figure 1). Debris had collected within this space. The left earlobe was normal and easily mobile. His right ear was normal. Deep tendon reflexes were normal.

**Figure 2:**
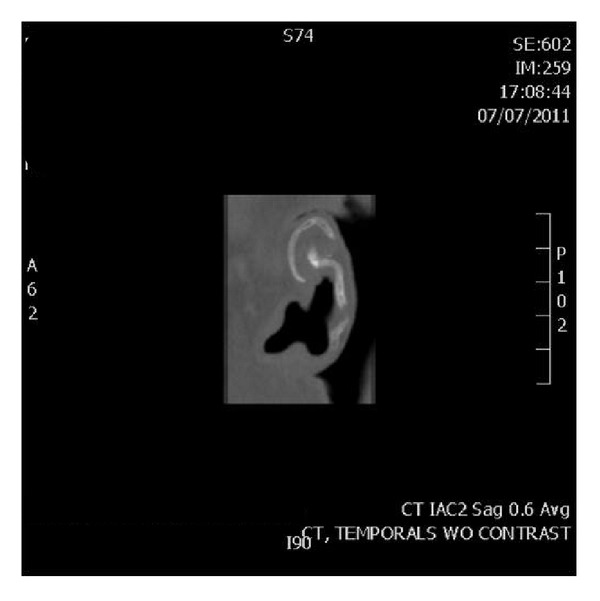
Computed tomography scan of left ear displays partial ossification of the external auricular cartilage.

A noncontrast temporal bone computed tomography (CT) scan demonstrated partial ossification of the left external auricular cartilage (Figure 2). Ossification was favored in this case due to the presence of tiny radiolucent air spaces seen within the opacity. Laboratory testing to include complete metabolic panel, serum calcium and phosphorus, parathyroid hormone, serum morning cortisol, adrenocorticotropic hormone, rapid plasma reagin, uric acid, hemoglobin A1c, vitamin D, and thyroid function tests was within the normal range. Complete blood count revealed a mild microcytic anemia. Based on these clinical and radiographic findings, a rare case of a petrified left ear was diagnosed.

## 3. Discussion

Bochdalek first reported a case of petrified auricle in a cadaver in 1866 [1], while Wassmund first reported the X-ray findings of this condition in 1899 [2]. Since then, approximately 140 cases of petrified ear have been reported with evidence of either calcification or ossification of the elastic auricular cartilage by radiography and/or histopathologic examination [3].

Elastic cartilage is a component of the auricle, external ear canal, nose, and epiglottis of the head and neck and does not usually have a tendency to calcify or ossify. Elastic cartilage of the auricle is normally highly malleable and painless to manipulate. Upon calcification or ossification of the auricle, it becomes difficult to maneuver and rock hard or petrified. Petrification of the auricular cartilage has been attributed to dystrophic calcification, metastatic calcification, and ectopic ossification. The petrification process can be initiated by local injury, such as frostbite, physical trauma, or as a result of systemic or inflammatory conditions. The most common cause of auricular calcification and ossification is frostbite [4, 5].

Petrification is caused more often by calcification than ossification [6]. Dystrophic calcification occurs when a patient has normal serum calcium and phosphorous levels, but calcium is deposited in previously damaged tissue. The auricle of the ear is vulnerable to local trauma and frostbite which can damage the auricular cartilage, result in calcium deposition, and lead to stiffening of the auricle [4, 7]. Metastatic calcification is due to an imbalance in calcium metabolism and occurs secondary to hypercalcemia, milk-alkali syndrome, vitamin D intoxication, hyperparathyroidism, and sarcoidosis [4, 8]. Adrenal insufficiency, leading to hypercalcemia, is the most common etiology of metastatic calcification of the auricle [4]. Jarvis et al. reported a high incidence of rigid ears in Addisonian patients [9]. It is believed that cortisol deficiency may contribute to the development of hypercalcemia in not only Addison's disease, but also in hypopituitarism and adrenogenital syndrome [10, 11]. Other systemic diseases that have been associated with auricular calcification include hypertension, alkaptonuria, systemic chondromalacia, familial cold hypersensitivity, relapsing polychondritis, scleroderma, polyarteritis nodosa, acromegaly, diabetes mellitus, hypothyroidism, hyperthyroidism, hyperparathyroidism, and pseudohyperparathyroidism [1, 5, 12–15].

Ectopic ossification occurs when new bone is formed in tissue that does not normally have a tendency to ossify. It can be classified as primary when it arises de novo, such as in rare syndromes, or as secondary if it occurs within an existing lesion and is often preceded by calcification [16]. True ossification of the auricle is a rare diagnosis, with less than 20 cases having been histopathologically documented [1, 3, 5, 12–14, 17–21]. It typically begins with the production of bone morphogenetic protein [6] which is necessary for the transformation of primitive cells to osteogenic precursor cells [3]. Besides frostbite, other local causes of ossification are recurrent cold exposure, mechanical trauma, repeated manipulation of the auricle, radiation therapy, acne scarring, and insect bites [21]. It has also been reported in association with inflammatory conditions such as chondritis, perichondritis, and syphilitic perichondritis [4, 7, 10]. Moreover, auricular ossification has been associated with benign melanocytic nevi, pilomatricomas, chondroid syringomas, and external auditory canal exostoses [13, 17] as well as with syndromes such as congenital plaque-like osteomatosis, Albright's hereditary osteodystrophy, fibrodysplasia ossificans progressiva, and osseous heteroplasia [13]. Collagen vascular diseases such as morphea, scleroderma, CREST, and childhood dermatomyositis can demonstrate areas of both calcification and ossification [12, 21].

The clinical presentation of petrified ear can vary, and there is no clinical difference between auricular calcification and ossification [22]. Most patients are asymptomatic, although some may experience discomfort with application of pressure, such as when sleeping on the affected ear. Bilateral involvement tends to be more frequent than unilaterality, and petrified ear seems to occur more commonly in men than in women [7]. On physical exam, the superior pinna is rigid and immobile. The ear lobule is spared. Rarely, patients have subjective and/or objective hearing loss which may be evaluated with an audiogram. External otalgia may be present if the process involves the ear canal.

Laboratory evaluation is helpful in detecting any underlying metabolic or endocrine disorder or other systemic disease. Laboratory analysis should include at a minimum a complete blood count, basic metabolic panel, thyroid function tests, parathyroid hormone levels, vitamin D, and serum calcium and phosphorus. If the fasting glucose is high, a hemoglobin A1c should be evaluated.

Further assessment should include radiography. A skull X-ray may demonstrate a hyperdense area, but a temporal bone CT can more specifically evaluate this finding. Calcification and ossification appear as hyperdense areas. Ossification is suspected by the presence of a trabecular bone pattern of minute radiolucent spaces within the dense opacities on CT [5, 19].

Histopathological examination is not mandatory but can confirm the diagnosis. It aids in the distinction between auricular calcification and ossification. 

Because most patients are asymptomatic, no specific therapy is necessary. The condition can be progressive, extending into the external ear canal cartilage, but it always spares the ear lobule. Evaluating for and correcting or controlling any underlying metabolic, endocrine, or other systemic condition is essential as well as protecting the affected auricle from further trauma. There is no known treatment to reverse the calcification or ossification. Patients who are symptomatic have been reported to show improvement with wedge resection of the affected cartilage or conchal reduction surgery [5, 18, 23]. Most asymptomatic patients choose to forgo surgery, as in our patient's case. 

Our patient declined auricular biopsy for histopathological confirmation as he understood the lack of available treatment for his mostly asymptomatic condition. He planned to follow up with audiology for adjustment of his hearing aid. 

Petrified ear has been associated with serious, potentially life-threatening, endocrine, and metabolic disorders, such as cortisol deficiency [5, 10, 11]. Its diagnosis warrants an investigation for cause, thereby eliminating delays in diagnosis of an underlying systemic condition. Patient morbidity could be minimized by recognizing petrified ear and expeditiously diagnosing and treating any related disorders.
